# Neutrophil Extracellular Traps Promote the Formation of Canine Dental Calculus

**DOI:** 10.3390/vetsci13060593

**Published:** 2026-06-18

**Authors:** Yufei Yang, Liwei Zeng, Ruizi Ren, Dongqiang Zheng, Yipeng Jin, Hao Shi

**Affiliations:** 1 College of Veterinary Medicine, China Agricultural University, Beijing 100193, China; 2 China Agricultural University Veterinary Teaching Hospital, Beijing 100193, China

**Keywords:** dog, dental calculus, neutrophil extracellular traps, mineralization, DNase I

## Abstract

Dental calculus is a highly prevalent oral condition in dogs and is widely recognized as an important risk factor for gingival inflammation and periodontal disease. Effective strategies for its prevention and treatment remain limited, highlighting the significance of exploring novel mechanisms underlying its formation. Neutrophil extracellular traps (NETs), a key component of innate immunity, have been found in various diseases. To investigate the relationship between NETs and canine dental calculus formation, NET-associated markers were assessed in the oral cavities of dogs with dental calculus and healthy controls. Based on previously published full-length 16S rRNA amplicon sequencing data of canine dental calculus, *Porphyromonas gulae* was selected as a candidate NET-inducing bacterium for subsequent validation experiments. Subsequent neutrophil stimulation experiments were conducted to explore the effects of NETs and related factors on dental calculus formation. Collectively, our findings demonstrate the presence of NETs within canine dental calculus and reveal that *P. gulae* present in canine dental calculus is capable of inducing NET formation. The level of myeloperoxidase–DNA complex in gingival crevicular fluid was significantly elevated in dogs with dental calculus. NETs promoted aggregation and microcrystal formation from calcium and phosphate ions under both physiological and supersaturated concentrations. By adhering to the surface of dental calculus, NETs facilitated calculus accumulation. This effect showed positive correlation with neutrophil counts and administration frequency, but was independent of the concentration of administered calcium and phosphate solutions. IL-1β promoted the formation of aggregated NETs but did not enhance calculus accumulation. DNase I inhibited this process by degrading NET-DNA. In conclusion, dental calculus and the calculus-inhabiting *P. gulae* could stimulate oral neutrophils to release NETs, which participate in and facilitate the initial formation, aggregation, and subsequent accumulation of canine dental calculus.

## Simple Summary

Dental calculus is one of the most common oral condition in dogs and is closely associated with gingivitis, periodontitis, tooth loss, and systemic health problems. However, the biological mechanisms underlying its formation are still not fully understood. In this study, we investigated whether neutrophil extracellular traps, which are web-like structures released by immune cells to capture microbes, participate in the development of canine dental calculus. We found that these structures were present in dental calculus samples from affected dogs and were significantly increased in the oral fluid of dogs with calculus compared with healthy dogs. The oral bacterium *Porphyromonas gulae*, which is commonly found in canine dental calculus, was able to stimulate neutrophils to release these extracellular structures. Further experiments showed that they promoted the formation, aggregation, and attachment of calcium-phosphate crystals, thereby accelerating calculus accumulation. Their effects became stronger with increased neutrophil numbers and repeated stimulation, while degradation of extracellular DNA reduced crystal accumulation and inhibited calculus enlargement. These findings demonstrate that immune responses are directly involved in canine dental calculus formation and suggest that targeting extracellular DNA or excessive neutrophil activation may provide new strategies for preventing and controlling dental calculus in dogs.

## 1. Introduction

Dental calculus, formed through mineralization of dental plaque biofilms, is a highly prevalent oral condition in dogs and an important risk factor for gingival and periodontal diseases. Epidemiological studies indicate that the incidence of canine dental calculus ranges from 40% to 100% across different age groups [1], and its prevalence and severity increase with age. Notably, over 80% of dogs aged above 12 years exhibit moderate-to-severe calculus deposition [1]. Dental calculus is considered an important risk factor for periodontal disease and is associated with halitosis [2], gingivitis [3] and tooth loss [4]. Furthermore, chronic periodontal inflammation has been associated with systemic disorders, including cardiovascular [5] and renal diseases [6], through bacteremia and persistent inflammatory responses. Thus, early prevention and intervention of dental calculus are critical for canine health, significantly reducing the incidence of severe complications and mortality.

Traditionally, dental calculus formation primarily results from the deposition of calcium and phosphate ions from saliva within dental plaque biofilms, influenced by multiple factors including bacteria, food debris, salivary physicochemical properties and local host factors [7,8]. Based on current knowledge, therapeutic strategies for canine dental calculus remain limited, particularly in surgical interventions. Recent studies have revealed that host immune responses may contribute to various human calculus formation processes through the release of neutrophil extracellular traps (NETs) [7,9,10,11]. However, the precise role of NETs in canine dental calculus pathogenesis and the factors stimulating NET production in the canine oral cavity remain unclear.

NETs are web-like structures released by neutrophils in response to different types of stimulation. They consist of a DNA scaffold decorated with histones and granular proteins such as myeloperoxidase (MPO) and neutrophil elastase (NE) [12]. The main function of NETs involves pathogen entrapment and antimicrobial activity. Excessive or sustained NET release may contribute to tissue damage and pathological calcification [13]. Studies in human gallstone formation have demonstrated that NETs promote the aggregation of calcium and cholesterol crystals, facilitating gallstone development [9]. Monosodium urate (MSU) crystals can induce neutrophils to form aggregated NETs (aggNETs), which encapsulate MSU crystals while degrading proinflammatory cytokines and providing the structural foundation for tophi formation, ultimately contributing to the pathogenesis of articular gout [14].

Various factors could stimulate neutrophils to release NETs, including microorganisms and their products, cytokines, immune complexes, autoantibodies, crystals, platelets, and chemical agents. Phorbol 12-myristate 13-acetate (PMA) is commonly used to induce NET formation in experimental settings. Multiple cytokines, including IL-6, TNF-α, and IL-1β, are critical inducers of NET formation [15]. IL-1β, a key proinflammatory cytokine implicated in periodontitis, can trigger inflammatory cascades and promote bone resorption at elevated concentrations [16]. Existing studies have reported conflicting findings regarding the role of IL-1β in NET formation. One study demonstrated that IL-1β enhanced monosodium urate crystal-induced aggNET formation but could not independently initiate NET release [14]. Conversely, another study reported that IL-1β alone could induce NET formation, with anti-IL-1β antibodies effectively reducing this response [17].

In human dental medicine, NETs have been conclusively demonstrated to accelerate plaque calcification [18]. Canine dental calculus shares similar inorganic constituents with human calculus [19]. However, the oral environment of dogs, characterized by alkaline saliva [8] and previously reported canine dental calculus-associated microbiota [20], may predispose to enhanced NET-mediated mineralization processes. Therefore, this study aims to investigate whether NETs are involved in and promote the formation of canine dental calculus, as well as to identify potential factors influencing NET generation and calculus development. The findings are expected to provide novel immunological insights into canine dental calculus pathogenesis and suggest new strategies for clinical prevention and treatment.

## 2. Materials and Methods

### 2.1. Animals

The study included 10 healthy dogs and 10 dogs with dental calculus, which were recruited from the China Agricultural University Veterinary Teaching Hospital. Detailed signalment information is provided in Supplementary Table S1. Gingival crevicular fluid (GCF) was collected from healthy dogs, while both GCF and dental calculus samples were obtained from affected dogs. Blood samples were collected from a separate cohort of healthy donor dogs owned by hospital staff for neutrophil isolation. All animals underwent comprehensive clinical examinations, basic dental evaluations, complete blood counts (CBC), and biochemical profiling to ensure the healthy dogs were free from oral and systemic diseases, with no history of antibiotic use within one month prior to the study. Dogs with dental calculus were further screened to exclude those with oral malignancies, severe systemic disorders, hemorrhagic diseases, acute infectious diseases, or any other conditions that would contraindicate supragingival or subgingival scaling procedures. The animal studies were approved by the Institutional Animal Care and Use Committee (IACUC) of China Agricultural University for Laboratory Animal Welfare and Ethical Review of Animal Experiments. The studies were conducted in accordance with the local legislation and institutional requirements.

### 2.2. Sample Collection and Processing

Dental calculus and gingival crevicular fluid were collected under general anesthesia. Prior to sampling, the target teeth were isolated using sterile gauze and dried with an air syringe. Sterile absorbent paper points were gently inserted along the buccal tooth axis into the gingival sulcus until slight resistance was encountered, maintained for precisely 30 s, then removed. The non-saturated portions of the paper points were aseptically trimmed, and the saturated segments were immediately transferred to sterile 1.5 mL microcentrifuge tubes. Any visibly blood-contaminated paper points were discarded to minimize contamination by circulating neutrophils and blood-derived MPO-DNA complexes, thereby ensuring that subsequent analyses reflected NET-associated markers present in GCF. Six replicates were obtained per animal. Dental calculus was maximally harvested using sterile hemostatic forceps. All collected calculus and GCF samples were rapidly frozen and stored at −80 °C. Peripheral blood samples (8–12 mL) were aseptically drawn from the posterolateral saphenous vein of healthy dogs and temporarily stored in sodium citrate anticoagulant tubes.

A 200 μL amount of sterile PBS containing 2 μL protease inhibitor phenylmethylsulfonyl fluoride (PMSF) was added to the microcentrifuge tubes. The paper points were then homogenized using a TissueMaster™ handheld tissue homogenizer (Beyotime, Shanghai, China), followed by centrifugation at 5000 rpm for 15 min at 4 °C. The supernatant was carefully collected for subsequent analysis.

### 2.3. Morphological Observation and Component Analysis of Canine Dental Calculus

The dental calculus samples were washed with distilled water, dried in an oven at 56 °C, and then examined for surface morphology using a stereomicroscope (Cossim, Beijing, China). Fourier transform infrared spectroscopy (FTIR) and energy-dispersive X-ray spectroscopy (EDS) were subsequently employed to analyze the major inorganic components of the canine dental calculus.

### 2.4. Immunofluorescence Staining

The dental calculus samples were fixed in 4% paraformaldehyde for 48 h and then decalcified in 10% EDTA solution (pH 7.2) for 3–4 days until complete decalcification (assessed by needle penetration). Decalcified specimens were paraffin-embedded and sectioned for detection of extracellular DNA (ecDNA), NE, and citrullinated histone H3 (citH3). After deparaffinization and antigen retrieval, sections were blocked with 5% bovine serum albumin (BSA) in PBS at 37 °C for 1 h. According to the manufacturer’s protocol, citH3 antibodies (Rabbit, ab1503, Abcam, Cambridge, UK, 1:200) or NE antibodies (Rabbit, bs-23549R, Bioss, Beijing, China, 1:200) were incubated overnight at 4 °C. Anti-rabbit fluorescent secondary antibodies (AS011, Abclonal, Wuhan, China, 1:100) were incubated at room temperature for 1 h away from light. The cell nucleus was re-dyed with propidium iodide (PI) reagent (C0080, Solarbio, Beijing, China, 1:100) at room temperature, away from light, for 10 min. Finally, the sealing solution of the anti-fluorescence quench agent was added to the slide, and the cover glass was placed. Image acquisition was performed on a confocal laser scanning microscope (A1HD25, Nikon, Tokyo, Japan) with NIS-Elements Viewer 5.2 software.

### 2.5. Detection of MPO-DNA Complexes in GCF by ELISA

According to the manufacturer’s protocol, the concentration of MPO-DNA complexes in GCF from both healthy dogs and those with dental calculus was quantitatively measured using a canine-specific ELISA kit (MM-85344O1, Meimian, Yancheng, China). Each sample was analyzed in three technical replicates, and the mean value was used for statistical analysis. The optical density (OD) of each well was measured at a wavelength of 450 nm. The OD value was converted to concentration (expressed in ng/L) based on the standard curve generated with the assay.

### 2.6. Neutrophil Stimulation Assays

#### 2.6.1. Isolation and Identification of Canine Peripheral Blood Neutrophils

Neutrophils were isolated from healthy canine venous blood using a canine-specific peripheral blood neutrophil isolation kit (LZS1087, TBDscience, Tianjin, China). Briefly, whole blood was carefully layered onto Separation Solution #2 and centrifuged at 800× *g* for 45 min at room temperature. After centrifugation, the plasma layer, mononuclear cell interface, and separation medium were carefully removed. The neutrophil-rich fraction above the erythrocyte layer was collected and subjected to red blood cell lysis. The purified neutrophils were finally resuspended in 1 mL of RPMI-1640 medium supplemented with 10% fetal bovine serum (FBS). Cell viability (>95%) was assessed by trypan blue exclusion, while purity (>95%) was determined by Wright–Giemsa staining.

#### 2.6.2. Establishment of NET Release Model

Cells suspended in RPMI-1640 medium supplemented with 10% FBS were seeded onto poly-L-lysine-coated coverslips placed in 24-well plates at a density of 5 × 10^5^ cells per well. Neutrophils were incubated at 37 °C for 30 min followed by stimulation with 500 nM phorbol 12-myristate 13-acetate (PMA) for 4 h to induce NET formation. Parallel experimental groups received either: (1) PMA (500 nM) + DNase I (100 U/mL; EN401-01, Vazyme, Nanjing, China) to enzymatically degrade NET-DNA, or (2) no treatment (untreated control). All conditions were maintained at 37 °C in a 5% CO_2_ humidified incubator.

#### 2.6.3. Identification of NET Release Model

##### Immunofluorescent Staining

To characterize NET formation, ecDNA and cit H3 were localized by immunofluorescence staining. Cell slides were fixed with 4% paraformaldehyde at room temperature for 20 min, followed by blocking with 5% BSA at 37 °C for 1 h. Primary antibody against cit H3 (Rabbit, ab1503, Abcam, 1:200) was incubated overnight at 4 °C. An anti-rabbit fluorescent secondary antibody (AS011, Abclonal, 1:100) was incubated at room temperature for 1 h in the dark. Nuclei were counterstained with propidium iodide (PI) reagent (C0080, Solarbio, China, 1:100) for 10 min in the dark at room temperatures. Finally, slides were mounted using an antifade mounting medium, and the cover glass was placed. Images were acquired using a fluorescence microscope (Olympus, Tokyo, Japan).

##### Western Blot

For quantitative analysis of NET formation, cit H3 levels were compared between control and PMA-treated groups using immunoblotting. After removing the culture medium, cells were lysed with 50–100 μL of RIPA buffer (containing protease inhibitors at 1:100 dilution of PMSF). An equal amount of protein was separated by SDS-PAGE and then transferred onto PVDF membranes (Millipore, MA, USA). The membranes were blocked with 5% non-fat milk and incubated overnight at 4 °C with primary antibodies against GAPDH (Mouse, Cat No. 60004-1-Ig, Proteintech, Wuhan, China, 1:5000) and cit H3 (Rabbit, ab1503, Abcam, 1:1000). After washing, the membranes were incubated with horseradish peroxidase (HRP)-conjugated secondary antibodies for 1 h at room temperature. The bands were visualized and imaged by an enhanced chemiluminescence (ECL) imaging system (Clinx Science Instruments Co., Ltd., Shanghai, China). The relative intensities of the bands were analyzed by ImageJ software (ImageJ version 1.46r).

#### 2.6.4. Stimulation Assays with *Porphyromonas gulae*

Based on previously published microbial sequencing data from canine dental calculus [20], *Porphyromonas gulae* was selected as a potential NET-inducing bacterium for subsequent validation experiments. The standard strain *Porphyromonas gulae* (ATCC 51700, *P. gulae*) was purchased from the American Type Culture Collection. Preliminary optimization experiments were conducted using *P. gulae* at multiplicity of infections (MOI) of 10, 100, and 1000 prior to the formal experiments. Based on the reproducibility and extent of NET formation observed by immunofluorescence, MOI 1000 was selected for subsequent studies. *P. gulae* was inoculated on 10% brain–heart infusion (BHI) sheep blood plates (Solarbio, Beijing, China) containing 5 μg/mL hemoglobin chloride, 1 μg/mL vitamin K1 (VK1) and 5 mg/mL yeast extract (Solarbio, Beijing, China). The incubation took place at 37 °C with 80% humidity under anaerobic conditions (10% CO_2_, 10% H_2_, and 80% N_2_) within an anaerobic culture vessel (YY-s Plus, LongFuJia Biotechnology, Beijing, China) for a culture period of 7–15 days. Single colonies were selected and re-streaked onto fresh blood agar plates to ensure culture purity before further experiments. The resulting single colonies were inoculated with a 5% fetal bovine serum (FBS) BHI liquid medium containing hemoglobin chloride and VK1 to be enriched. *P. gulae* were grown to the logarithmic growth phase for 48 h. The optical density at 600 nm of the bacterial cell suspensions was measured spectrophotometrically to estimate bacterial numbers, and the bacteria were heat-killed at 80 °C for 30 min. Neutrophils were stimulated with the positive controls PMA (500 nM) and *P. gulae* (MOI, 1000) after being equilibrated for a 30 min baseline period. Unstimulated neutrophils were employed as negative controls. The ability of *P. gulae* to stimulate neutrophils to produce NETs was qualitatively detected by immunofluorescence imaging, as previously described.

### 2.7. Mineral Ion Crystallization Assays

Based on compositional analysis of canine dental calculus, culture media containing major mineral ions were prepared with two concentration groups: physiological (2 mM CaCl_2_·2H_2_O + 1.2 mM K_2_HPO_4_) and supersaturated (5 mM CaCl_2_·2H_2_O + 3 mM K_2_HPO_4_), with pH adjusted to 8.0. In 24-well plates, 5 × 10^5^ neutrophils were seeded in 1 mL RPMI-1640 supplemented with 10% FBS. Three experimental groups were established: (1) untreated control, (2) PMA-treated (500 nM), and (3) PMA (500 nM) + DNase I (100 U/mL), with triplicate wells per group. After 4 h incubation (37 °C, 5% CO_2_), culture medium was replaced with mineral solutions: the physiological group received 1 mL each of 2 mM CaCl_2_·2H_2_O and 1.2 mM K_2_HPO_4_, while the supersaturated group received 1 mL each of 5 mM CaCl_2_·2H_2_O and 3 mM K_2_HPO_4_ (final pH 8.0 for both). Following 3-day incubation (37 °C), crystal formation was analyzed using bright-field microscopy and stereomicroscopy, with three independent experimental replicates performed.

### 2.8. Dental Calculus–NET Adhesion Assays

The dental calculus samples underwent sequential processing including ultrasonic cleaning (70 kHz, 20 min) and autoclaving (121 °C, 20 min), followed by 24 h DNase I treatment (100 U/mL) to eliminate background DNA. In 24-well plates, 5 × 10^5^ neutrophils were cultured in 1 mL RPMI-1640 with 10% FBS under three experimental conditions: (1) untreated control, (2) PMA (500 nM), and (3) PMA (500 nM) + DNase I (100 U/mL), with triplicate wells per group. After 30 min pre-incubation (37 °C, 5% CO_2_), calculus specimens were added to all wells and cultured for 6 h with hourly gentle agitation. Post-incubation, medium was replaced with either DNase I solution (100 U/mL) or deionized water (control) for overnight treatment. Following PI staining (10 μg/mL, 15 min, RT, dark), ecDNA on calculus surfaces was immediately visualized using a Nikon SMZ18 stereomicroscope with three biological replicates.

### 2.9. Dental Calculus Mineralization Assays

The dental calculus samples underwent sequential processing including ultrasonic cleaning (70 kHz, 20 min) and autoclaving (121 °C, 20 min). Samples were then dehydrated in a precision oven at 56 °C for 5 h to achieve constant mass. Dry weight was measured and recorded using an analytical balance with an accuracy of 0.0001 g.

#### 2.9.1. The Effect of NETs on Mineralization of Canine Dental Calculus

In 24-well plates, 5 × 10^5^ neutrophils were seeded in 1 mL RPMI-1640 supplemented with 10% FBS. Three experimental groups were established: (1) untreated control, (2) PMA (500 nM), and (3) PMA (500 nM) + DNase I (100 U/mL), with six biological replicates per group. After 30 min pre-incubation (37 °C, 5% CO_2_), dental calculus specimens were introduced for 6 h co-culture. Post-incubation, DNase I (Group 3) or deionized water (controls) was added overnight. The medium was replaced with mineralization solution (1 mL 2 mM CaCl_2_·2H_2_O + 1 mL 1.2 mM K_2_HPO_4_, pH 8.0) for 14-day culture, with three intermittent NET administrations.

As with the above groupings, the medium was replaced with mineralization solution (1 mL 2 mM CaCl_2_·2H_2_O with 25 μM calcein and 1 mL 1.2 mM K_2_HPO_4_, pH 8.0) for 3 days in darkness. After PI staining (10 μg/mL, 15 min, RT), calculus surfaces were examined by fluorescence stereomicroscopy (Nikon SMZ18) to visualize ecDNA and calcium deposition.

#### 2.9.2. The Effect of Neutrophil Count on Mineralization of Canine Dental Calculus

In 24-well plates, neutrophils were seeded in 1 mL RPMI-1640 supplemented with 10% FBS at different densities to evaluate the effect of neutrophil count on dental calculus mineralization. Three experimental groups were established: (1) 2.5 × 10^5^ neutrophils/well, (2) 5 × 10^5^ neutrophils/well and (3) 1 × 10^6^ neutrophils/well. Each group comprised six replicates (three untreated control wells and three wells treated with 500 nM PMA). After 30 min pre-incubation (37 °C, 5% CO_2_), dental calculus specimens were introduced for 6 h co-culture. The cell culture medium was then discarded, and 1 mL of 2 mM CaCl_2_·2H_2_O and 1 mL of 1.2 mM K_2_HPO_4_ (pH 8.0) were added to each well, followed by continued culture for 14 days.

#### 2.9.3. The Effect of NET Administration Frequency on Mineralization of Canine Dental Calculus

In 24-well plates, 1 × 10^6^ neutrophils and 500 nM were added in 1 mL RPMI-1640 supplemented with 10% FBS. Two experimental groups were established: (1) received NETs three times during culture; (2) received NETs once during culture, with each group comprising ten replicates. After 30 min pre-incubation (37 °C, 5% CO_2_), dental calculus specimens were introduced for 6 h co-culture. The cell culture medium was then discarded, and 1 mL of 2 mM CaCl_2_·2H_2_O and 1 mL of 1.2 mM K_2_HPO_4_ (pH 8.0) were added to each well, followed by continued culture for 14 days.

#### 2.9.4. The Effect of Mineral Ion Concentration and NETs on Mineralization of Canine Dental Calculus

A 1 mL amount of RPMI-1640 supplemented with 10% FBS was added to each well of 24-well plates. Three experimental groups were established: (1) 2.5 × 10^5^ neutrophils/well, (2) 5 × 10^5^ neutrophils/well and (3) 1 × 10^6^ neutrophils/well, with each group comprising six replicates. Then, 500 nM PMA was added in each well. After 30 min pre-incubation (37 °C, 5% CO_2_), dental calculus specimens were introduced for 6 h co-culture. The cell culture medium was then discarded. In each experimental group, three wells were treated with 1 mL of 2 mM CaCl_2_·2H_2_O and 1 mL of 1.2 mM K_2_HPO_4_, while the other three wells received 1 mL of 5 mM CaCl_2_·2H_2_O and 1 mL of 3 mM K_2_HPO_4_ per well, followed by continued culture for 14 days.

#### 2.9.5. The Effect of IL-1β on PMA-Induced NET Formation and Mineralization of Canine Dental Calculus

Cells in RPMI with 10% FBS were seeded onto polylysine-coated cell sheets in 24-well plates at a density of 1 × 10^6^ cells. Neutrophils were incubated at 37 °C for 30 min. Three experimental groups were established: (1) untreated control, (2) PMA (500 nM), (3) IL-1β (50 ng/mL) and (4) PMA (500 nM) + IL-1β (50 ng/mL). After culturing at 37 °C and 5% CO_2_ for 6 h, cit H3 and ecDNA in different groups were detected by an immunofluorescence assay as above.

In 24-well plates, 1 × 10^6^ neutrophils were added to 1 mL RPMI-1640 supplemented with 10% FBS. Two experimental groups were established: (1) PMA (500 nM) and (2) PMA (500 nM) + IL-1β (50 ng/mL). After 30 min pre-incubation (37 °C, 5% CO_2_), dental calculus specimens were introduced for 6 h co-culture. The cell culture medium was then discarded, and 1 mL of 2 mM CaCl_2_·2H_2_O and 1 mL of 1.2 mM K_2_HPO_4_ (pH 8.0) were added to each well, followed by continued culture for 14 days.

After the experiment, the dental calculus was weighed again using an electronic balance and the results were recorded. The percentage weight gain of dental calculus (%) for each group was calculated as follows: Percentage weight gain (%) = (Post-culture dental calculus weight − Pre-culture dental calculus weight)/Pre-culture dental calculus weight × 100%.

### 2.10. Statistical Analysis

Results are expressed as mean ± standard deviation (SD) from at least three biological replicates calculated on the average of at least two technical replicates. Statistical analysis of results was performed using GraphPad Prism version 10. Samples exhibiting normal distribution were analyzed for statistical significance using independent *t*-tests, while those with non-normal distribution were assessed by Wilcoxon rank-sum tests. A *p*-value of <0.05 was considered statistically significant.

## 3. Results

### 3.1. Surface Morphology of Canine Dental Calculus

**Figure 1:**
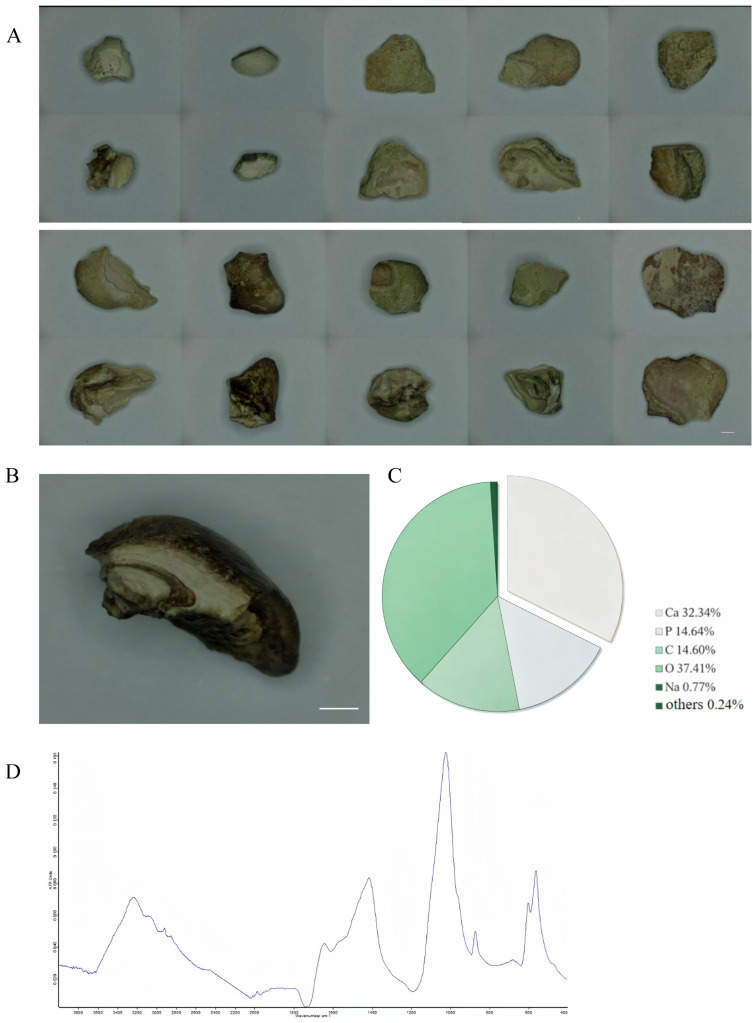
Morphological and compositional analysis of canine dental calculus. (**A**) Representative images of isolated canine dental calculus samples collected from tooth surfaces, showing the free and tooth-contacting surfaces. Scale bars, 1 mm. (**B**) Representative image showing the layered structure of an isolated canine dental calculus sample. Scale bars, 1 mm. (**C**) Elemental analysis of canine dental calculus. (**D**) Infrared spectrum of canine dental calculus.

The free surface of canine dental calculus is directly exposed to the oral environment, making it susceptible to food, saliva, and bacterial influences, resulting in complex surface morphology. Typically exhibiting an irregular, uneven, and rough texture, it appears yellowish-white, brown to black, with color intensity potentially dependent on calculus composition and formation duration (Figure 1A). The margins are irregular, presenting as jagged or flake-like. The attached surface maintains close contact with tooth enamel, showing more uniform mineral deposition that yields a relatively smooth and flat surface, though with minor undulations. While similar in color to the free surface, its edges are more distinct, tightly adapted to the tooth contour with a curved profile, appearing denser and harder due to enamel contact. Stratification with varying colors is often observable on the attached surface, with cross-sections sometimes revealing a core structure (Figure 1B), demonstrating that calculus formation is a dynamic, continuous process where eliminating contributing factors may help mitigate further progression.

### 3.2. The Presence of NETs in Canine Dental Calculus

Scanning electron microscopy and EDS revealed that calcium and phosphorus were among the predominant elements in canine dental calculus. The elemental composition shown in Figure 1C represents the mean values obtained from 10 independent tests. Fourier-transform infrared spectroscopy showed characteristic peaks at approximately 566, 603, and 1028 cm^−1^. The bands at 566 and 603 cm^−1^ corresponded to CaO vibration modes, while distinct absorption bands between 1200 and 800 cm^−1^ and 670–500 cm^−1^ were observed due to PO_4_^3−^ ion vibrations in hydroxyl-deficient phosphates. The absorption near 1028 cm^−1^ was associated with PO_4_^3−^, and within this broad absorption band (peak at 1028 cm^−1^), a characteristic principal band appeared at 872 cm^−1^ attributable to CO_3_^2−^, with the strongest peak near 1415 cm^−1^ also related to CO_3_^2−^ (Figure 1D). These findings indicate that the primary inorganic component of canine dental calculus is carbonated hydroxyapatite.

**Figure 2:**
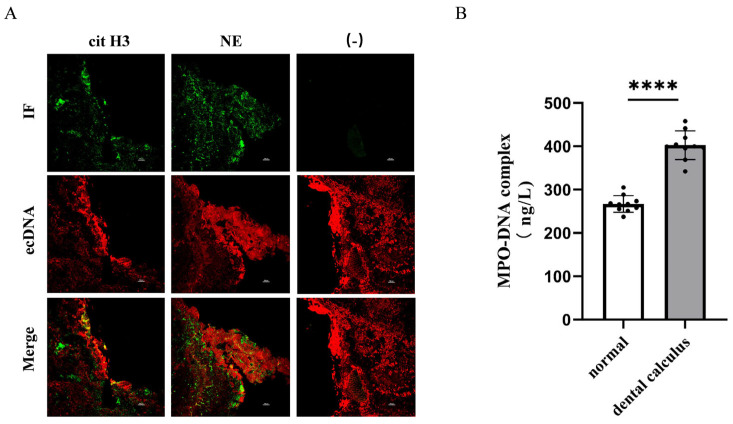
Evidence of neutrophil extracellular traps (NETs) in canine dental calculus. (**A**) CLSM images taken from canine dental calculi. Co-localization of NET markers (Merge), including cit H3 (green), NE (green) and extracellular DNA (ecDNA, red), was apparent in canine dental calculus. Scale bars, 100 μm. (**B**) ELISA of the myeloperoxidase–DNA (MPO–DNA) complexes further confirmed higher NET content in the gingival crevicular fluids (GCF) of dogs with dental calculus deposits. Each dot represents the mean value of each sample. Statistical significance was determined between the two groups of dogs (*n* = 10). Data represent mean ± s.d. *p* values were determined using two-sided Student’s *t*-test. **** *p* < 0.0001.

Immunofluorescence results demonstrated the presence of NET markers in canine dental calculus (Figure 2A). NETs originated from neutrophils in GCF (Figure 2B), which was supported by significantly higher MPO-DNA concentrations in calculus-affected dogs (402.51 ± 32.97 ng/L) compared to healthy controls (267.03 ± 19.16 ng/L), *p* < 0.0001. These observations confirm the presence of NETs in canine dental calculus.

### 3.3. Validation of NET Release Model

**Figure 3:**
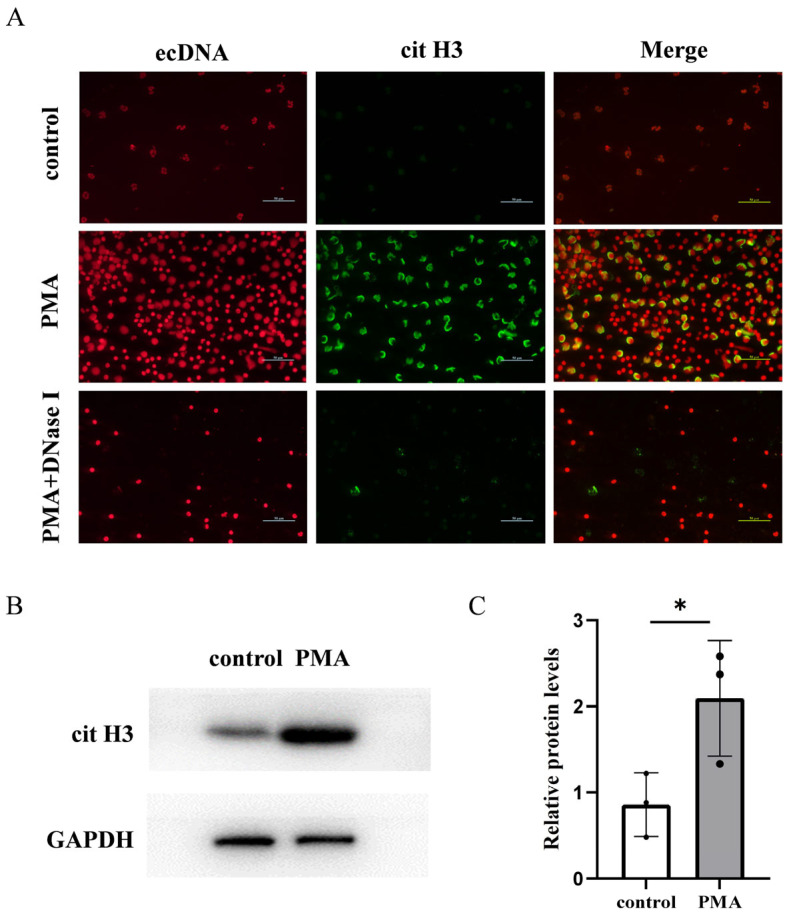
Establishment of NET release model in vitro. (**A**) Immunofluorescence images taken from canine neutrophils. PMA-treated neutrophils showed colocalization of citH3 (green) and ecDNA (red) while the other groups did not, confirming NET formation in the group treated with PMA. Control, untreated group; PMA, group treated with PMA (500 nM); PMA + DNase I, group treated with PMA (500 nM) and DNase I (100 U/mL). Scale bars, 50 μm. (**B**,**C**) Western blotting confirmed that PMA-treated neutrophils produced more cit H3. * *p* < 0.05.

The immunofluorescence image revealed that neutrophils stimulated by PMA exhibited overlapping fluorescence signals between ecDNA and cit H3, whereas the PMA + DNase I treatment group exhibited attenuated fluorescence signals (Figure 3A). Western blot analysis demonstrated significantly elevated cit H3 protein expression levels in the PMA-treated group compared to the control group (*p* < 0.05) (Figure 3B,C). These results indicate the successful establishment of an in vitro NET model and confirm the effective degradation of PMA-induced NETs by DNase I.

### 3.4. Neutrophils Release NETs as a Response to P. gulae

**Figure 4:**
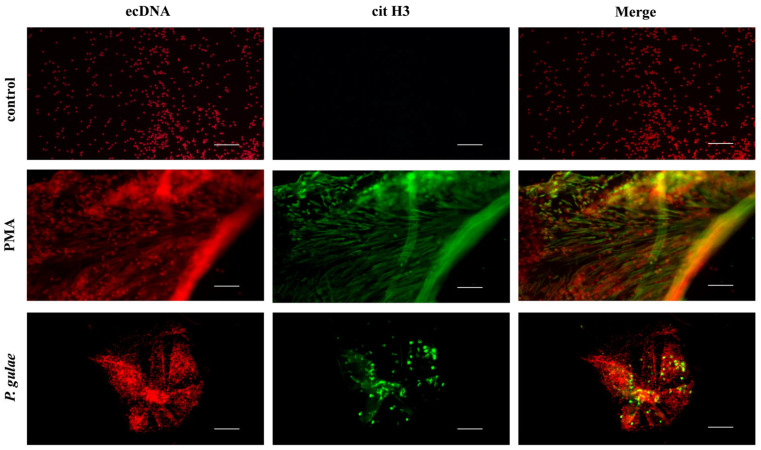
Neutrophils release NETs as a response to *P. gulae*. Colocalization of ecDNA and cit H3 was observed in both the PMA-treated and *P. gulae*-treated groups, whereas little cit H3 staining was detected in untreated controls. PMA stimulation induced extensive web-like extracellular structures positive for both ecDNA and cit H3, consistent with robust NET formation. Similar NET morphology was observed in *P. gulae*-treated neutrophils. Control, untreated group; PMA, group treated with PMA (500 nM); *P. gulae*, group treated with *P. gulae* (MOI 1:1000). Scale bars, 100 μm.

The immunofluorescence image revealed that neutrophils stimulated by *P. gulae* exhibited overlapping fluorescence signals between ecDNA and cit H3 (Figure 4), indicating that *P. gulae* possesses the ability to induce NET formation. However, compared to the PMA-treated group, the co-localization areas of NET markers were relatively limited.

### 3.5. NETs Promote Mineral Ion Crystallization

**Figure 5:**
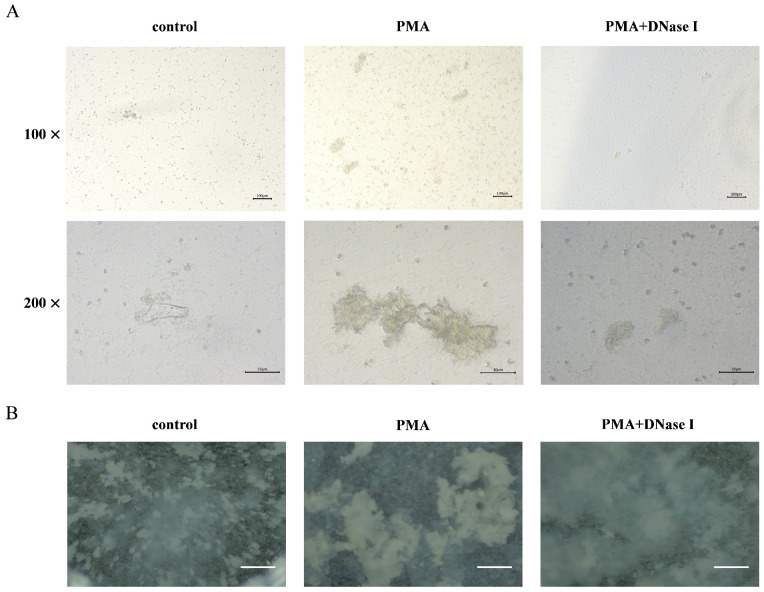
The results of mineral ion crystallization assay. (**A**) Crystallization in the physiological concentration (2 mM CaCl_2_·2H_2_O + 1.2 mM K_2_HPO_4_, pH 8.0) group under inverted microscopy. (**B**) Crystal aggregation in the supersaturated concentration (5 mM CaCl_2_·2H_2_O + 3 mM K_2_HPO_4_, pH 8.0) group under stereomicroscopy. Scale bars, 1 mm.

Under physiological concentrations of calcium and phosphate ions, the control group showed minimal crystal formation, while the PMA group not only developed crystals but also exhibited significant crystal aggregation. However, DNase I treatment markedly reduced both crystal formation and aggregation (Figure 5A). In supersaturated calcium-phosphate solutions, the control group displayed scattered granular crystals, whereas the PMA group formed large crystal clusters. DNase I treatment partially degraded these clusters, resulting in a mist-like dispersion (Figure 5B). These findings demonstrate that NETs can not only induce crystal nucleation and aggregation from physiological ion concentrations but also promote the clustering of crystals precipitated from supersaturated solutions.

### 3.6. NETs Promote Dental Calculus–NET Adhesion

**Figure 6:**
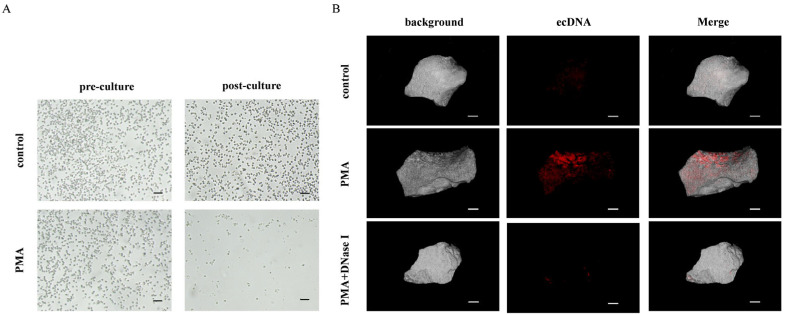
The results of dental calculus–NET adhesion assays. (**A**) Changes in the number of neutrophils after the administration of PMA and canine dental calculus. Scale bars, 50 μm. (**B**) Surface-adhered ecDNA on canine dental calculus visualized by fluorescence stereomicroscopy. PMA-treated neutrophils showed ecDNA while the other groups did not, confirming NET formation in group treated with PMA. Control, untreated group; PMA, group treated with PMA (500 nM); PMA + DNase I, group treated with PMA (500 nM) and DNase I (100 U/mL). Scale bars, 1 mm.

**Figure 7:**
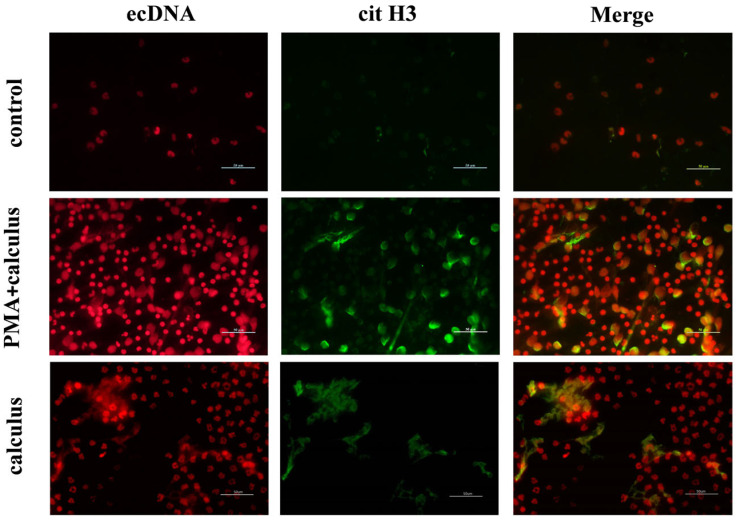
Immunofluorescence identification of NET formation stimulated by canine dental calculus. Neutrophils release NETs when exposed to canine dental calculus alone for 6 h. Compared to the PMA + calculus group, most neutrophils in the group treated for calculus maintain normal nuclear morphology without chromatin decondensation, and fewer NETs are released. Control, untreated group; calculus, group treated for calculus; PMA + calculus, group treated with PMA (500 nM) and calculus. Scale bars, 50 μm. EcDNA is shown in red and cit H3 is shown in green.

Compared to the control group, the PMA group showed a significant reduction in neutrophil counts in cell culture dishes following the addition of canine dental calculus (Figure 6A), while untreated neutrophils neither adhered to the calculus surface nor released sufficient NETs to bind to the surface upon stimulation (Figure 7). Stereomicroscopic fluorescence imaging revealed markedly increased ecDNA on the calculus surface in the PMA group compared to controls, with subsequent degradation observed after DNase I treatment (Figure 6B). These results demonstrate that NETs can adhere to canine dental calculus surfaces, whereas neutrophils themselves cannot, and that DNase I effectively degrades calculus-bound NET-DNA.

### 3.7. NETs Promote Dental Calculus Mineralization

**Figure 8:**
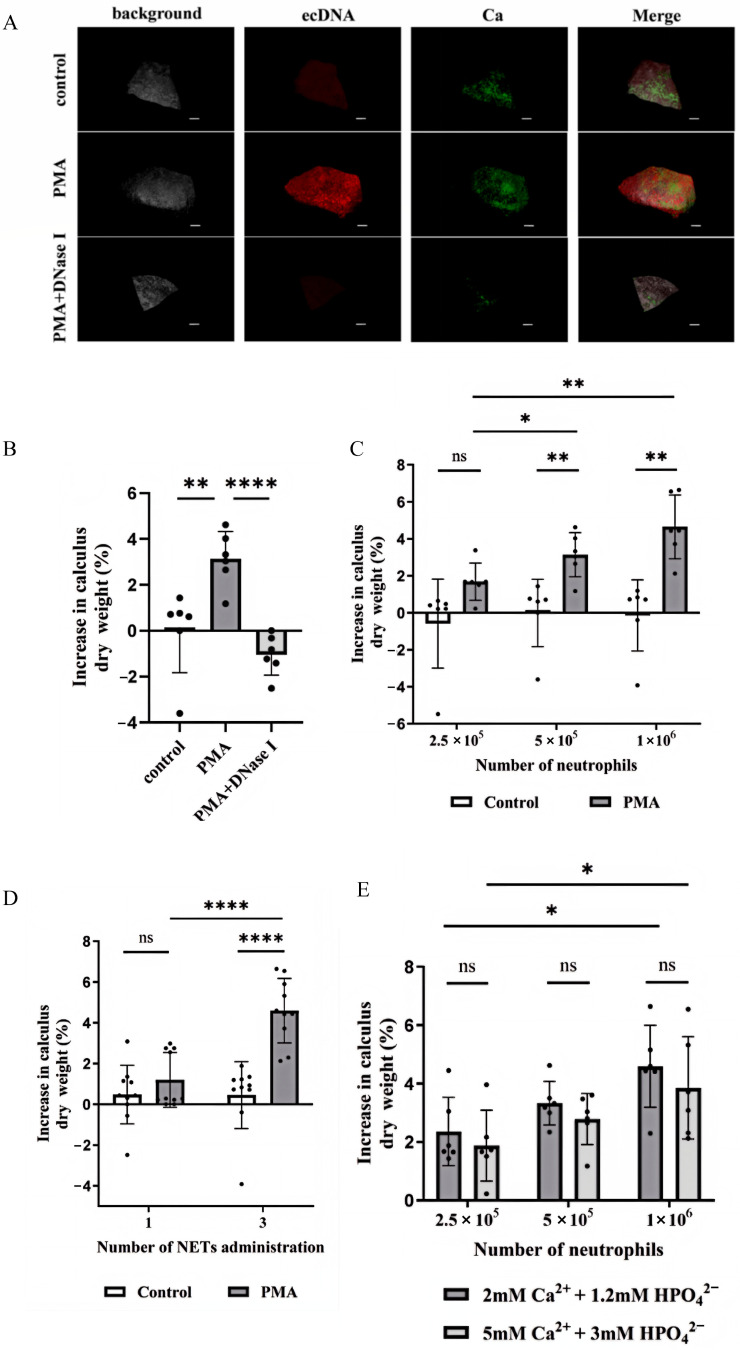
The results of dental calculus mineralization assays. (**A**) Fluorescent stereomicroscopy images taken from canine dental calculi. PMA-treated neutrophils showed colocalization of ecDNA and calcified nidi, while the other groups did not. Control, untreated group; PMA, group treated with PMA (500 nM); PMA + DNase I, group treated with PMA (500 nM) and DNase I (100 U/mL). Scale bars, 1 mm. (**B**) Effect of NETs on mineralization of canine dental calculus (*n* = 6). (**C**) Effect of neutrophil count on mineralization of canine dental calculus (*n* = 6). (**D**) Effect of NET administration frequency on mineralization of canine dental calculus (*n* = 10). (**E**) Effects of mineral ion concentration and NETs on mineralization of canine dental calculus (*n* = 6). * *p* < 0.05, ** *p* < 0.01, **** *p* < 0.0001; ns, not significant.

We administered 5 × 10^5^ neutrophils/mL three times over a two-week period, with concurrent PMA stimulation. Stereomicroscopic fluorescence microscopy revealed substantial ecDNA adhesion to rough calculus surfaces with concomitant calcium deposition in the PMA group compared to controls. DNase I treatment effectively degraded surface-bound ecDNA and markedly reduced calcium deposition (Figure 8A). As shown in Figure 8B, the PMA group exhibited significantly higher percentage weight gain (3.14 ± 1.19%) than the control group (−0.14 ± 1.92%) (*p* < 0.01). The PMA + DNase I group showed a significantly lower weight gain (−1.05 ± 0.90%) than the PMA group (*p* < 0.0001). These findings support a role for NETs in canine dental calculus accumulation.

Calculus weight gain increased with neutrophil quantity and administration frequency. No significant difference was observed between the two calcium-phosphate solution concentrations tested (Figure 8C–E). However, this result only indicates that the two concentrations tested produced similar effects under the present experimental conditions.

**Figure 9:**
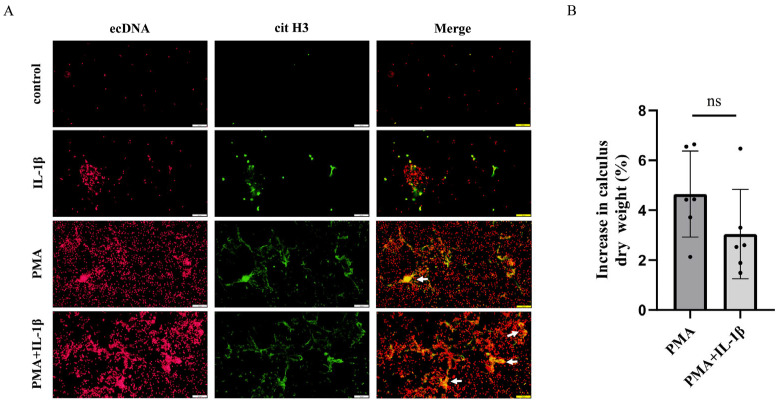
Effect of IL-1β on NET formation and mineralization of canine dental calculus. (**A**) Immunofluorescence identification of NETs induced by IL-1β and PMA in neutrophils. More aggNETs (arrows) formed in PMA + IL-1β group. Scale bars, 100 μm. (**B**) The effect of combination of PMA and IL-1β on weight gain of canine dental calculus (*n* = 6). Control, untreated group; IL-1β, group treated with IL-1β (50 ng/mL); PMA, group treated with PMA (500 nM); PMA + IL-1β, group treated with PMA (500 nM) and IL-1β (50 ng/mL). EcDNA is shown in red and cit H3 is shown in green. ns, not significant.

IL-1β treatment induced marked neutrophil aggregation. The PMA + IL-1β groups showed increased aggNET formation compared to PMA groups (Figure 9A). However, after three NET administrations and two weeks of culture, the percentage weight gain in PMA + IL-1β groups (3.05 ± 1.73%) showed no statistically significant difference from that in PMA groups (4.65 ± 1.75%) (Figure 9B).

## 4. Discussion

Morphological examination revealed layered structures in canine dental calculus, and corresponding layered distributions of ecDNA were observed within calculus sections. In addition, MPO-DNA concentrations in GCF were significantly higher in dogs with dental calculus than in healthy controls, confirming the presence of NETs and suggesting that periodontal neutrophils may represent an important source of these structures. The parallel distribution of ecDNA and calculus layers suggests that NETs may participate in the progressive accumulation of canine dental calculus. Because NET formation is driven by neutrophil activation, persistent gingival inflammation and continuous neutrophil infiltration in severe periodontal disease may create conditions favorable for sustained NET release [21]. Once released, NETs may serve as additional scaffolds for calcium-phosphate deposition and thereby facilitate the progressive accumulation of dental calculus.

NET formation has been reported in periodontal lesions and is considered an important component of host responses to periodontal pathogens [22]. Our findings do not exclude the possibility that NETs arise secondary to inflammation in vivo. However, our in vitro experiments demonstrated that calcium-phosphate crystal nucleation, aggregation, and mineral accumulation were enhanced in the presence of NET structures, whereas these effects were partially reduced following DNase I treatment. Therefore, our findings suggest that NETs are not merely a consequence of inflammation but may also actively contribute to the mineralization process associated with canine dental calculus formation.

Bacteria serve as key triggers for NET formation in the oral cavity. Previous studies investigating the microbial composition of canine dental calculus have identified *P. gulae* as one of the predominant bacterial species and a major pathogen associated with canine periodontal disease [20,23]. Studies on *P. gingivalis*, a congeneric species, have demonstrated its ability to induce NET formation through LPS-mediated activation of the Ca^2+^-TPL2-MEK-ERK-PAD4 signaling pathway [24]. Although these findings provide useful insights into the potential interaction between *Porphyromonas* species and neutrophils, the NET-inducing capacity of *P. gulae* has not previously been reported. In the present study, we demonstrated that *P. gulae* was capable of inducing NET formation in canine neutrophils. However, whether the underlying mechanisms are similar to those reported for *P. gingivalis* remains unknown and warrants further investigation.

Dental calculus is a common oral condition not only in dogs but also in cats and humans. However, substantial interspecies differences exist in oral microbial communities and the pathogenesis of dental calculus formation [25,26]. In dogs, *Porphyromonas* spp. are considered key periodontal pathogens and are frequently detected in dental plaque and calculus-associated biofilms [27]. Human periodontal disease is more commonly associated with *P. gingivalis*, *Tannerella forsythia*, and *Treponema denticola* [28]. Therefore, mechanisms identified in human studies may not be directly applicable to dogs, highlighting the importance of investigating canine-specific pathways involved in dental calculus formation.

EcDNA appears to play a central role in this process. The negatively charged ecDNA functions as an anionic scaffold that attracts and stabilizes calcium ions, providing nucleation sites for calcium-phosphate deposition and promoting mineralization [13]. Apoptosis-generated ecDNA can similarly interact with extracellular matrices to form nucleation sites [29]. In addition, NETs form web-like extracellular structures capable of binding microorganisms [12], and these structures may similarly facilitate the retention of mineral particles on existing calculus surfaces, potentially contributing to progressive calculus accumulation. Sterilized canine dental calculus was able to induce modest NET release in our study, suggesting that once calculus is formed, it may further promote NET formation and contribute to continued mineral accumulation. Nevertheless, the in vivo process is likely more complex and may also involve interactions among extracellular DNA, NET-associated proteins, and other components of the periodontal microenvironment [30,31].

Factors influencing NET formation may similarly affect dental calculus accumulation. In vitro mineralization assays demonstrated that the extent of NET-promoted calculus weight gain correlated with neutrophil quantity and administration frequency, but showed no dependence on the two tested calcium-phosphate solution concentrations. These findings suggest that inhibiting NET formation or reducing availability of NET-derived extracellular components may represent a potential strategy to control dental calculus progression.

Contrary to previous study suggesting that higher calcium-phosphate ion availability promotes dental calculus formation by facilitating calcium-phosphate deposition [32], our results did not support this hypothesis. This discrepancy may arise from the following factors: (1) The simplified in vitro environment lacks complex salivary proteins and has limited quantities of neutrophils/NETs, which may be insufficient to precipitate additional calcium ions. Increasing NET levels might yield different outcomes [33]. (2) While we confirmed that NETs can aggregate pre-formed calcium-phosphate crystals, the underlying mechanisms remain unclear. It is currently unknown whether calculus-adherent NETs directly bind existing crystalline clusters, recruit calcium ions from unsaturated ionic solutions, or retain sufficient adhesive functionality after aggregation to facilitate attachment to dental calculus surfaces. Prolonged culture and repetitive handling may also cause crystal cluster detachment from calculus surfaces. These factors collectively explain the absence of significant weight differences between the two mineral concentration groups.

Notably, these conclusions derive from sterile in vitro conditions distinct from the complex oral environment. Clinical applicability requires validation through in vivo studies. Future research could enhance reliability by expanding in vitro test groups, refining concentration gradients, increasing sample sizes, and conducting animal experiments. Moreover, the relationship between NETs and the formation of other types of canine calculi also will be explored in the future.

The role of IL-1β in NET formation remains controversial. While some research indicates IL-1β cannot independently induce NET release [14], our results demonstrated that IL-1β promoted both neutrophil aggregation and NET formation. Sequential treatment with IL-1β followed by PMA generated significantly more aggNETs than PMA alone, whereas the reverse treatment sequence did not produce a similar effect. This finding suggests that the primary contribution of IL-1β may be the recruitment and accumulation of neutrophils, thereby creating conditions favorable for subsequent NET and aggNET formation. Previous studies have shown that NETs can modulate IL-1β release from immune cells including neutrophils and macrophages [17,34,35]. IL-1β may further amplify local inflammatory responses and neutrophil recruitment [36,37]. Although aggNETs can resolve inflammation, they also promote pathological calcifications like tophi [38]. In our study, comparable calculus weight gain between PMA + IL-1β and PMA groups suggests that increased aggNET formation did not result in greater mineral accumulation under the experimental conditions used. Nevertheless, the potential contribution of IL-1β to dental calculus progression in vivo cannot be excluded and warrants further investigation.

From a clinical perspective, these findings suggest that dental calculus formation is not solely a passive physicochemical process but may also involve active host immune responses. Excessive neutrophil activation and NET release may therefore represent previously underappreciated contributors to calculus accumulation. In addition to routine plaque control, strategies aimed at reducing chronic gingival inflammation or modulating excessive NET formation may help slow calculus progression [35,39,40]. Furthermore, NET-associated biomarkers such as MPO-DNA complexes in GCF may have value for monitoring periodontal inflammation. Their potential utility as biomarkers associated with dental calculus susceptibility warrant further investigation.

Several limitations should be considered when interpreting the findings of this study. NET formation induced by *P. gulae* was evaluated primarily by immunofluorescence staining, and additional quantitative assays were not performed. Neutrophil viability following bacterial stimulation was also not assessed. Although NETs were identified based on the co-localization of ecDNA and cit H3, future studies incorporating quantitative analyses and viability measurements would provide a more comprehensive assessment of neutrophil responses. In addition, dental plaque samples from healthy dogs were not included for comparison. Because the primary objective of this study was to investigate the involvement of NETs in canine dental calculus formation, NET-associated changes in healthy plaque were not examined. Comparative analysis of plaque samples from healthy and calculus-affected dogs may help further elucidate the transition from dental biofilm to mineralized calculus. Moreover, the mineralization experiments were conducted using a simplified in vitro model and therefore may not fully recapitulate the complexity of the canine oral and periodontal environment.

Nevertheless, the present findings support a potential role for NETs in canine dental calculus formation. The detection of NET-associated components within dental calculus, together with the promotive effect of NETs on calcium-phosphate deposition in vitro, suggests that NETs may contribute to the mineralization process. Further investigation in larger canine populations, particularly through longitudinal studies, will help clarify the relationships among dental plaque accumulation, periodontal disease progression, NET formation, and dental calculus development, and may provide a basis for exploring NET-targeted approaches to dental calculus prevention.

## 5. Conclusions

Dental calculus and the calculus-inhabiting *P. gulae* could stimulate oral neutrophils to release NETs. NETs participate in and facilitate the initial formation, aggregation, and subsequent accumulation of canine dental calculus, while DNase I exerts inhibitory effects during this process. Reducing neutrophil counts and NET administration frequency can mitigate progressive calculus weight gain. This study provides novel immunological insights into canine calculus pathogenesis and suggests potential strategies for clinical prevention and treatment.

## Data Availability

The original contributions presented in this study are included in the article. Further inquiries can be directed to the corresponding authors.

## Supplementary Materials

The following supporting information can be downloaded at: https://www.mdpi.com/article/10.3390/vetsci13060593/s1, Table S1: Signalment of dogs included in this study. Table S2: Relative citH3/GAPDH ratios obtained from three independent experiments. Relative citH3 protein expression levels were normalized to GAPDH. Figure S1: Original Western blot exposure image of cit H3 (~17 kDa) protein detected with anti-cit H3 antibody. M indicates molecular weight markers; PMA indicates the PMA-treated group; C indicates the untreated control group. The image shown represents the original exposure file retained from the experiment. Figure S2: Original Western blot exposure image of GAPDH (~36 kDa), used as the loading control. M indicates molecular weight markers; PMA indicates the PMA-treated group; C indicates the untreated control group. The image shown represents the original exposure file retained from the experiment.

## Abbreviations

The following abbreviations are used in this manuscript:

NETs	Neutrophil Extracellular Traps
MPO	Myeloperoxidase
NE	Neutrophil Elastase
MSU	Monosodium urate
aggNETs	Aggregated NETs
GCF	Gingival crevicular fluid
CBC	Complete blood counts
PMSF	Phenylmethylsulfonyl fluoride
PBS	Phosphate-buffered saline
FTIR	Fourier transform infrared spectroscopy
EDS	Energy-dispersive X-ray spectroscopy
DNA	Deoxyribonucleic Acid
ecDNA	Extracellular DNA
DNase I	Deoxyribonuclease I
PMA	Phorbol 12-Myristate 13-Acetate
PAD4	Peptidylarginine deiminase 4
cit H3	Citrullinated Histone H3
OD	Optical density
DAPI	4’,6-diamidino-2-phenylindole
