# Supplementary material for: Neutrophil Extracellular Traps Promote the Formation of Canine Dental Calculus
Source: Vet Sci. 2026 Jun 18;13(6):593. doi: 10.3390/vetsci13060593 (PMC13307829; doi:10.3390/vetsci13060593)
Supplement: Supplementary file 1 [file vetsci-13-00593-s001.zip › vetsci-4357617-Supplementary Materials.pdf]

Supplementary Materials

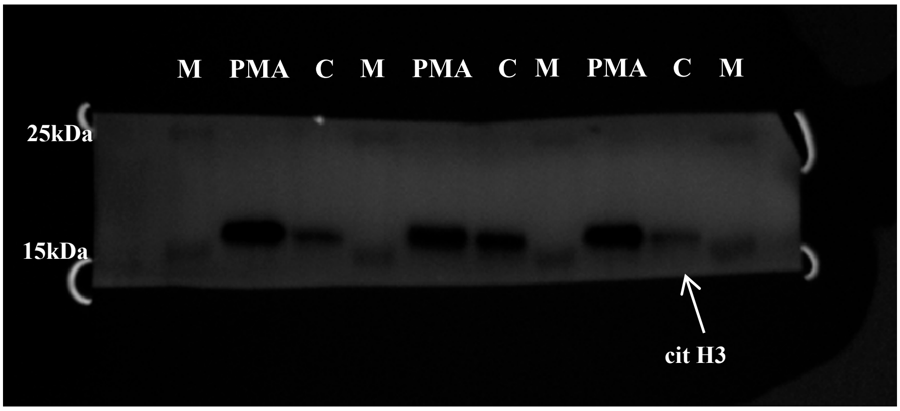

Figure S1. Original Western blot exposure image of cit H3 (~17 kDa) protein detected with anti-cit H3 antibody. M indicates molecular weight markers; PMA indicates the PMA-treated group; C indicates the untreated control group. The image shown represents the original exposure file retained from the experiment.

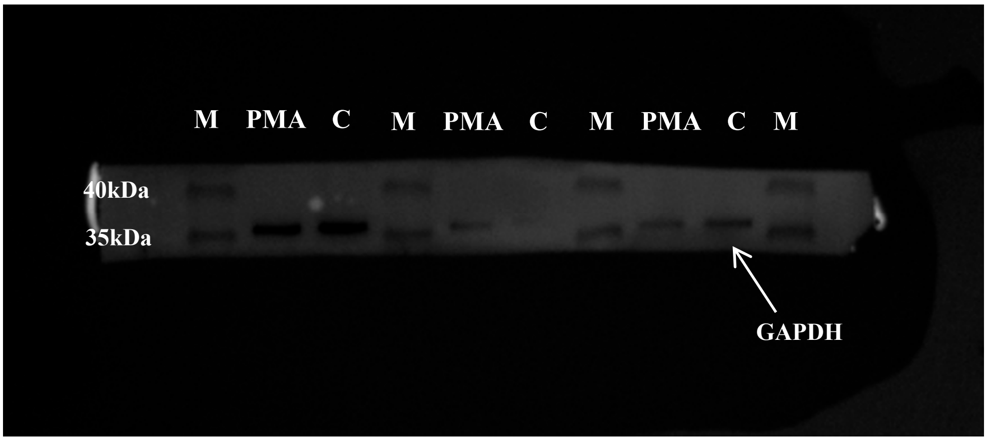

Figure S2. Original Western blot exposure image of GAPDH (~36 kDa), used as the loading control. M indicates molecular weight markers; PMA indicates the PMA-treated group; C indicates the untreated control group. The image shown represents the original exposure file retained from the experiment.

control	PMA
0.88	2.58
1.22	2.37
0.48	1.33

Table S2. Relative citH3/GAPDH ratios obtained from three independent experiments. Relative citH3 protein expression levels were normalized to GAPDH.
